# Task-Dependent Reorganization of Ankle–Knee Mechanical Coordination Revealed by Moment–Moment Phase Space Analysis

**DOI:** 10.3390/jfmk11020201

**Published:** 2026-05-19

**Authors:** Alessandro Garofolini, William Anthony Sparrow, Rezaul Begg

**Affiliations:** Institute for Health and Sport (IHES), Victoria University, Melbourne, VIC 8001, Australia; williamtony.sparrow@vu.edu.au (W.A.S.); rezaul.begg@vu.edu.au (R.B.)

**Keywords:** ankle–knee coordination, gait biomechanics, phase space analysis, joint moments, locomotion, motor control, coordination topology

## Abstract

**Background:** Human locomotion requires coordinated torque production across multiple joints, yet conventional gait analysis typically evaluates joint behavior independently, limiting insight into inter-joint coordination. This study aimed to quantify task-dependent reorganization of ankle–knee mechanical coordination using a moment–moment phase space framework. **Methods:** A normative dataset of healthy adults (*N* = 50) performing natural-speed walking, toe walking, heel walking, stair ascent, and stair descent was analyzed. Sagittal-plane external ankle and knee moments were extracted from time-normalized stride cycles and z-score normalized within each stride to emphasize coordination topology. Ankle–knee trajectories were represented in moment–moment space and characterized using three geometric metrics: loop magnitude (|Area|), principal axis orientation, and anisotropy. Metrics were aggregated within subject and analyzed using linear mixed-effects models with planned contrasts against walking. **Results:** Loop magnitude differed significantly across tasks (*p* < 0.001), with the largest increases observed during toe walking (+3.45 relative to walking) and stair descent (+2.41). Principal axis orientation also showed a significant task effect (*p* = 0.026), with stair descent producing the largest rotation of the coordination axis (−29.8°). Anisotropy varied significantly across tasks (*p* < 0.001), indicating systematic changes in the dimensionality and strength of inter-joint torque coupling. **Conclusions:** Locomotor tasks induce structured, task-dependent reorganization of ankle–knee coordination topology. Moment–moment phase space analysis provides a compact and interpretable framework for quantifying inter-joint torque coupling, with potential applications in biomechanics research and the development of activity-aware assistive technologies.

## 1. Introduction

Human locomotion emerges from coordinated torque production across multiple joints rather than isolated joint actions. During walking and stair negotiation, the ankle and knee work in unison to provide limb support, propulsion, and energy transfer, through dynamically evolving external moments [1]. Despite this inherently distributed mechanical organization, conventional gait analysis typically evaluates joint kinematics and kinetics independently, focusing on peak magnitudes, timing, or waveform characteristics [2]. Although such measures describe individual joint behavior, they provide limited insight into how joint torques co-evolve as an integrated mechanical system. Approaches that explicitly examine relationships between joints are therefore required to capture the coordinated mechanical structure of locomotion.

Inter-joint coordination changes in response to task demands. In locomotor control toe walking, heel walking, and stair ascent or descent impose constraints that redistribute joint torques. For example, stair ascent increases extensor work and vertical center-of-mass displacement, whereas stair descent emphasizes controlled energy absorption and braking [3,4,5]. Similarly, constraining foot posture modifies distal leverage that affects the relative mechanical contributions of ankle plantarflexors and knee extensors [6,7]. Because the lower limb functions as a mechanically coupled system, such changes in task mechanics necessarily induce coordinated adjustments across joints rather than independent modulation of individual joint torques. This perspective is consistent with motor control frameworks that emphasize coordination across redundant degrees of freedom [8,9,10], yet such coordination is rarely quantified directly at the level of joint torques.

Phase space representations of joint moments provide a principled framework for capturing the mechanical organization of inter-joint coordination. By plotting ankle external moments against knee external moments across the stride, a closed trajectory is formed in moment–moment space that reflects the joint evolution of distal limb torque production. Similar representations have been used to characterize coordination in kinematic domains through angle–angle diagrams and cyclograms [11,12,13], as well as through dimensionality reduction approaches that quantify coordination structure and variability [14,15]. Extending these approaches to joint moments provides a more direct link to underlying mechanical interactions, as joint moments reflect the net effect of muscle forces and external loads acting on the limb [16]. The topology of the resulting trajectory can be quantified using complementary geometric descriptors: loop magnitude reflects the overall extent of inter-joint torque exchange, principal axis orientation describes the dominant direction of torque co-variation, and anisotropy indicates the dimensionality of the coordination structure. The aim of this study was therefore to quantify task-dependent reorganization of ankle–knee mechanical coordination using moment–moment phase space analysis from a normative dataset of healthy individuals during level walking, toe walking, heel walking, and ascending and descending stairs.

## 2. Materials and Methods

### 2.1. Participants and Dataset

This study used a publicly available kinematic, kinetic, and electromyographic dataset of human locomotion acquired from 50 healthy individuals performing multiple locomotor tasks [17]. Full details regarding instrumentation, acquisition procedures, marker placement, preprocessing, and biomechanical modeling are reported in the original dataset publication [17]. The dataset included level walking trials performed at progressively varied speeds, as well as toe walking, heel walking, stair ascent (StepUp), and stair descent (StepDown) tasks. During the walking trials, participants initially walked at their natural-speed and subsequently performed trials at progressively faster and slower speeds without externally imposed cadence constraints. Toe walking, heel walking, and stair negotiation tasks were performed barefoot at self-selected speed. Stair ascent and descent were performed using a custom two-step staircase instrumented with a force platform beneath the first step [17]. Joint moments were computed using a standard biomechanical model and normalized to body mass. Sagittal-plane external joint moments for the ankle and knee were extracted from time-normalized strides (101 samples representing 0–100% of stride duration). To isolate task-dependent coordination independent of gait speed, self-selected speeds ±10% of the participant’s median were classified as natural-speed and retained for analysis. Toe walking, heel walking, stair ascent, and stair descent trials did not require speed selection. All analyses were performed in MATLAB (version R2026a, MathWorks, Natick, MA, USA).

### 2.2. Construction of Ankle–Knee Moment–Moment Phase Space

To reveal coordination structure rather than absolute magnitude the sagittal-plane external ankle and knee joint moments were extracted and joint moment trajectories z-score normalized within a stride:

(1) $$\tilde{M}(t)=\frac{M(t)-\mu }{\sigma }$$

where *M*(*t*) is the time-normalized moment trajectory, and *μ* and *σ* represent the mean and standard deviation across strides. This normalization is a common approach for comparing coordination patterns independent of signal amplitude and scaling [10,18]. Ankle moments were plotted against knee moments across the stride to generate a closed trajectory in moment–moment phase space. This representation captures the joint evolution of distal limb torque production independent of stride timing.

### 2.3. Quantification of Coordination Topology

Three scalar descriptors were derived from each stride’s moment–moment trajectory. These metrics were selected to capture complementary aspects of coordination structure, including the magnitude of inter-joint mechanical exchange, the dominant direction of joint moment co-variation, and the geometric dimensionality of coordination patterns.

#### 2.3.1. Loop Magnitude (|Area|)

The signed area enclosed by the closed ankle–knee trajectory was computed using the polygon (shoelace) method. The absolute value of the area was used as a measure of the magnitude of inter-joint mechanical exchange:

(2) $$|A|=|\frac{1}{2}\sum_{i=1}^{N}\left(x_{i}y_{i+1}-x_{i+1}y_{i}\right)|$$

where *x* and *y* correspond to the normalized ankle and knee moments. Larger loop areas therefore reflect greater redistribution of joint moments throughout the stride cycle.

#### 2.3.2. Principal Axis Orientation

Principal component analysis (PCA) was applied to the two-dimensional moment cloud (ankle vs. knee) for each stride to identify the dominant direction of joint moment co-variation, an approach commonly used to characterize coordination structure in multivariate biomechanical signals [14]. PCA was selected because it provides a compact and interpretable description of the dominant direction of covariance within multivariate biomechanical signals [14]. PCA was performed using the covariance matrix of the stride-wise normalized moment trajectories, computed across all time points within the stride. The orientation of the first principal component (PC1), representing the dominant coordination axis, was computed as:

(3) $$\theta =\mathrm{atan}2(v_{y},v_{x})$$

where $v_{x}$ and $v_{y}$ are the components of the eigenvector associated with the largest eigenvalue of the covariance matrix. Because the coordination axis is directionally symmetric (i.e., 0° ≡ 180°), PC1 orientations were treated as axial variables and mapped to the range 0–180°. To quantify task-induced rotation relative to natural walking, each participant’s PC1 orientation during a given task was expressed relative to their natural-speed walking orientation using axial angular differences constrained to ±90°.

#### 2.3.3. Shape Anisotropy

**Figure 1:**
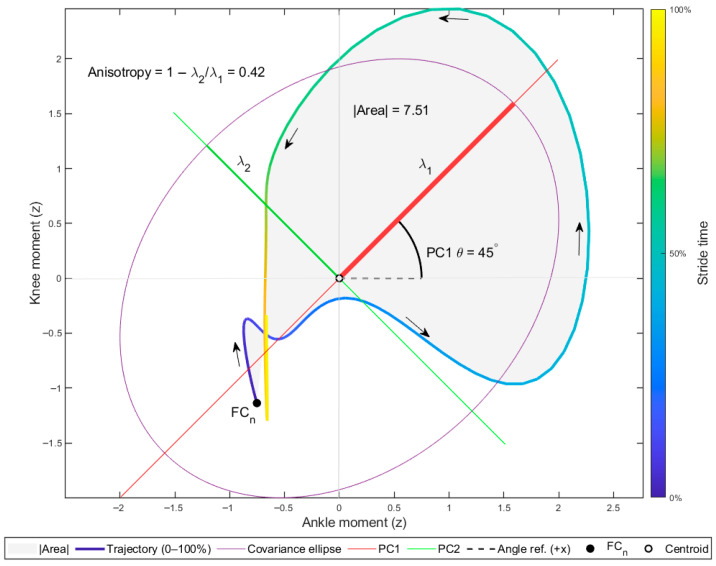
Conceptual representation of moment–moment phase space metrics used to quantify ankle–knee mechanical coordination. Sagittal-plane ankle and knee joint moments were z-score normalized within stride and plotted against each other to form a closed trajectory in moment–moment space. The loop area (|Area|) quantifies the magnitude of inter-joint torque exchange across the stride. Principal component analysis (PCA) of the moment cloud defines the dominant coordination axis (PC1; red line) and orthogonal secondary axis (PC2; green line), with eigenvalues *λ*_1_ and *λ*_2_ describing the variance along each direction. Loop anisotropy was computed as $1-\lambda_{2}/\lambda_{1}$, indicating the degree to which coordination is constrained to a linear relationship. The covariance ellipse illustrates the second-order structure of the moment distribution. Trajectory color indicates normalized stride time (0–100%). The normalized foot-contact point (FC_n_) and loop centroid are shown for reference. The black arrows indicate the direction of progression of the moment–moment trajectory throughout the normalized stride cycle (0–100% stride time).

The degree of directional dominance in the moment-moment loop was quantified using an anisotropy index derived from the PCA eigenvalues:

(4) $$\mathrm{Anisotropy}=1-\frac{\lambda_{2}}{\lambda_{1}}$$

where $\lambda_{1}$ and $\lambda_{2}$ are the first and second eigenvalues of the covariance matrix ($\lambda_{1}\geq \lambda_{2}$). Values approaching 1 indicate strongly elongated, linear coupling, whereas values approaching 0 indicate more circular coordination patterns. This metric therefore reflects the extent to which coordination is constrained along a dominant coupling axis versus distributed across multiple dimensions. A schematic representation of the moment–moment loop metrics is shown in Figure 1.

Additional geometric descriptors of loop compactness and trajectory validity, including circularity and loop closure error, are described in the Appendix A.

### 2.4. Aggregation

Stride-level metrics were computed for all included strides. To reduce within-subject variability and ensure independence of observations, metrics were aggregated within subject and task using the median across strides.

### 2.5. Statistical Analysis

Statistical analyses were conducted in MATLAB (version R2026a, MathWorks, Natick, MA, USA). For each subject and locomotor task, stride-level metrics were aggregated using the median across strides to obtain a single representative value per subject and condition. Task effects on loop magnitude (|Area|), anisotropy (1 − λ_2_/λ_1_), and principal axis orientation relative to natural walking (ΔAngle) were assessed using linear mixed-effects models with Task as a fixed factor and Subject included as a random intercept to account for repeated measures:

(5) Metric∼Task+(1∣Subject)

Separate models were fitted for each coordination metric. Model significance was evaluated using F-tests with Satterthwaite approximated degrees of freedom. When significant task effects were detected, planned pairwise contrasts comparing each locomotor task against natural walking were performed. *p*-values for these comparisons were adjusted using the Holm correction to control the family-wise error rate. Statistical significance was set at α = 0.05. Effect estimates and 95% confidence intervals are reported for all pairwise contrasts.

## 3. Results

### 3.1. Sample and Task Distribution

**Figure 2:**
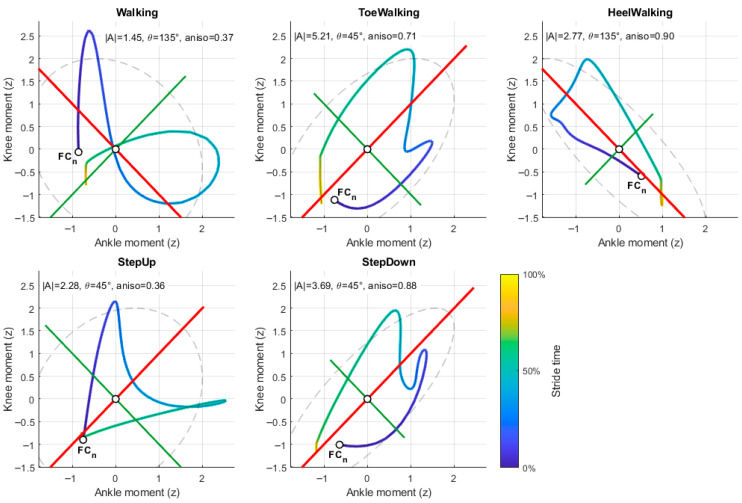
Representative ankle–knee moment–moment trajectories for each locomotor task. Example stride trajectories for walking, toe walking, heel walking, stair ascent (StepUp), and stair descent (StepDown). Joint moments were normalized within stride and plotted in ankle–knee phase space. The principal coordination axis (PC1; red line) and secondary axis (PC2; green line) are derived from PCA of the moment cloud. The dashed ellipse represents the covariance ellipse associated with the joint moment distribution. Trajectory color indicates normalized stride time (0–100%). For each example, the loop magnitude (|Area|), PC1 orientation (θ), and anisotropy (1 − λ_2_/λ_1_) are reported. These examples illustrate task-dependent differences in coordination topology.

Following natural-speed filtering, a total of 1102 strides were retained across five locomotor tasks: natural-speed walking (*n* = 331), toe walking (*n* = 278), heel walking (*n* = 178), stair ascent (*n* = 169), and stair descent (*n* = 146). Task-level sample characteristics are summarized in Table 1. Metrics were aggregated at the subject level using the median across strides prior to statistical analysis. Representative ankle–knee moment–moment trajectories for each task are illustrated in Figure 2.

### 3.2. Loop Magnitude (|Area|)

**Figure 3:**
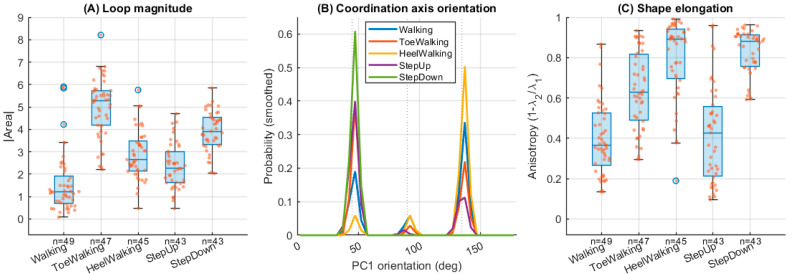
Task-dependent differences in ankle–knee coordination metrics. (**A**) Loop magnitude (|Area|) across locomotor tasks. Larger values indicate greater inter-joint torque exchange during the stride. (**B**) Distribution of principal axis orientations across tasks, illustrating clustering around two dominant coordination families (~45° and ~135°). The vertical dotted lines indicate angular reference orientations at 45°, 90°, and 135°. (**C**) Shape anisotropy (1 − λ_2_/λ_1_) reflecting the degree of linear coupling between ankle and knee moments. Points represent subject-level medians across strides, and boxplots summarize the distribution across participants. Toe walking and stair negotiation exhibit larger loop magnitudes compared to natural walking, while anisotropy varies across tasks, reflecting changes in the dimensionality of ankle–knee torque coordination.

Loop magnitude differed significantly across locomotor tasks (F(4,222; Satterthwaite) = 73.78, *p* < 0.001, Table 2). Planned contrasts against natural walking revealed that all locomotor tasks produced significantly larger loop magnitudes (all Holm-corrected *p* < 0.001). The largest increases were observed during toe walking (Δ = 3.45, 95% CI [3.01, 3.88]) and stair descent (Δ = 2.41, 95% CI [1.96, 2.85]), followed by heel walking (Δ = 1.35, 95% CI [0.92, 1.79]) and stair ascent (Δ = 0.81, 95% CI [0.37, 1.26]). As illustrated in Figure 3A, natural walking exhibited the smallest phase space loops, whereas toe walking and stair negotiation produced substantially larger trajectories, indicating increased inter-joint torque exchange between the ankle and knee.

### 3.3. Principal Axis Orientation (Relative to Walking)

Task effects on the principal coordination axis orientation relative to walking were significant (F(4,218; Satterthwaite) = 2.83, *p* = 0.026, Table 2). Planned contrasts showed that only stair descent differed significantly from walking after Holm correction (Δ = −29.8°, 95% CI [−48.3°, −11.4°], *p* = 0.0066). Toe walking, heel walking, and stair ascent did not differ significantly from walking. As shown in Figure 3B, coordination axis orientations clustered around two dominant families near ~45° and ~135°, indicating that ankle–knee torque coupling generally followed one of two preferred alignment directions across tasks. However, stair descent produced the most consistent deviation from the walking reference orientation.

### 3.4. Shape Anisotropy

Anisotropy differed significantly across locomotor tasks (F(4,222; Satterthwaite) = 54.87, *p* < 0.001, Table 2). Planned contrasts indicated that toe walking, heel walking, and stair descent all exhibited significantly higher anisotropy than walking (all Holm-corrected *p* < 0.001), indicating more elongated, line-like torque coupling patterns. In contrast, stair ascent did not differ significantly from walking (*p* = 0.56). Specifically, anisotropy increased during toe walking (Δ = 0.24, 95% CI [0.17, 0.32]), heel walking (Δ = 0.40, 95% CI [0.33, 0.48]), and stair descent (Δ = 0.44, 95% CI [0.36, 0.51]). As illustrated in Figure 3C, these tasks exhibited more elongated moment–moment trajectories compared to walking.

### 3.5. Summary of Task Effects

Together, these findings demonstrated that specific locomotor tasks systematically reorganise ankle–knee moment coordination, with changed distal posture or increased mechanical demands producing more open coordination plots due to increased inter-joint torque exchange. Meanwhile, stair descent uniquely induced a significant rotation of the dominant coordination axis, and several tasks exhibited increased anisotropy, indicating stronger linear coupling between ankle and knee moments. Mixed-effects model results are summarized in Table 2, and planned contrasts comparing each task to walking are reported in Table 3.

## 4. Discussion

### 4.1. Task-Dependent Reorganization of Coordination Topology

The present findings demonstrate that ankle–knee coordination exhibits structured, task-dependent reorganization according to mechanical and functional demands, rather than reflecting a single invariant coordination pattern across locomotor conditions. Across locomotor conditions, systematic changes were observed in loop magnitude, orientation, and anisotropy, indicating that inter-joint torque coupling adapts in a structured and predictable manner. These results extend prior work emphasizing the distributed nature of locomotor control [1,19] by showing that such coordination is not only distributed but geometrically reorganized in a low-dimensional state space. Recent work has increasingly emphasized that locomotor control reflects adaptive coordination across joints and segments shaped by task and environmental demands [20,21]. The present findings provide a compact representation of these adaptations, capturing how coordination structure reorganizes as a function of task-specific mechanical constraints.

### 4.2. Coordination as a Geometric Object Rather than a Waveform Property

A central implication of the present results is that inter-joint coordination should be conceptualized as a geometric object in phase space, rather than as a collection of waveform features. While conventional analyses focus on peak values, timing, or waveform similarity, the moment–moment loop provides a compact representation of how joint torques co-evolve throughout the stride. Such approaches characterize individual joint behavior over time but provide limited insight into how joint moments co-evolve as an integrated coordination structure throughout the stride. The observation that coordination loops exhibit task-specific changes in orientation and shape indicates that coordination is not invariant across conditions, even when overall movement patterns appear similar. This is consistent with dynamical systems perspectives on motor behavior [9], but also aligns with more recent work emphasizing low-dimensional structure and task-relevant variability in human movement [22,23]. From a dynamical systems perspective, locomotor behavior has long been recognized to exhibit nonlinear and adaptive properties, including structured variability and sensitivity to task constraints [24]. In parallel, the uncontrolled manifold (UCM) framework has demonstrated that movement variability is not random noise but is organized relative to task-relevant dimensions [25]. The present findings are consistent with these perspectives, but extend them by characterizing the geometry of inter-joint coordination directly in joint-moment phase space. Rather than quantifying variability alone, the proposed framework captures how coordination topology itself reorganizes across locomotor tasks. Importantly, variability in these representations is not merely noise but reflects functional organization and control strategies [25,26]. Recent advances in the study of locomotor modularity further support the view that movement can be described using reduced-dimensional coordination structures [27]. The moment–moment phase space extends this perspective by grounding these low-dimensional structures in mechanically meaningful variables, namely joint moments, thereby linking coordination geometry directly to the forces shaping movement.

### 4.3. Mechanical Demands Shape Coordination Geometry

The systematic modulation of loop geometry across tasks suggests that coordination structure is strongly shaped by mechanical demands. Tasks such as toe walking and stair negotiation, which impose increased distal constraints or energetic requirements, produced larger loop magnitudes, reflecting greater inter-joint torque exchange. From a neuromechanical perspective, enlarged loop magnitude may reflect increased redistribution of mechanical contributions across joints in response to changing stability, energetic, and propulsion demands. This is consistent with contemporary neuromechanical analyses showing that changes in locomotor conditions redistribute mechanical work across joints [6,28]. At the same time, changes in anisotropy indicate that tasks differ in the dimensionality of coordination. Increased anisotropy during constrained tasks suggests stronger alignment of joint torques along a dominant coordination axis, potentially reflecting tighter mechanical coupling under increased task demands. Conversely, more isotropic loops may indicate greater flexibility in how torque contributions are distributed across joints, consistent with contemporary perspectives on motor abundance, self-organization, and flexible coordination [9,25]. In this context, anisotropy can be interpreted as a geometric indicator of how strongly coordination is constrained along dominant neuromechanical coupling patterns. The rotation of the principal coordination axis observed during stair descent further highlights that the direction of torque co-variation is task-dependent, reflecting shifts in the relative contribution of proximal and distal joints. Such adaptations are consistent with the need for controlled energy absorption and stability during downward progression [5], but also align with recent findings that locomotor control strategies are dynamically reweighted across joints depending on stability and energetic requirements [29].

### 4.4. From Joint-Level Descriptions to Coordination-Level Representations

Traditional gait analysis has largely focused on joint-specific variables, implicitly assuming that coordination patterns are preserved across tasks [2]. The present results extend this perspective by demonstrating that inter-joint coordination undergoes systematic, task-dependent reorganization that can be quantified geometrically in moment–moment phase space. This interpretation is consistent with prior work emphasizing self-organization, motor abundance, and adaptive variability in locomotor control [9,24,25,30], but further suggests that such adaptations can be represented as changes in coordination topology. This shift toward coordination-level representations aligns with recent efforts in biomechanics and motor control to move beyond joint-centric descriptions and instead characterize movement using low-dimensional coordination structures [31,32]. The present framework extends these approaches by describing coordination directly in mechanically meaningful joint-moment space, thereby linking low-dimensional coordination geometry with the underlying forces governing locomotor behavior. Similar approaches have been applied in studies of muscle synergies and whole-body coordination, suggesting that movement control may operate in reduced-dimensional spaces that capture functional organization [27,33]. By representing coordination as a trajectory in moment–moment space, the present framework provides a mechanically interpretable and low-dimensional description of locomotor organization, bridging traditional biomechanics and contemporary control-based perspectives. In this sense, coordination topology offers a unifying representation linking joint mechanics, variability structure, and task constraints within a single analytical framework.

### 4.5. Implications for Assistive Technologies and Adaptive Control

The observed task-dependent reorganization of coordination may have translational relevance for assistive technologies and clinical gait assessment. Recent advances in exoskeleton and prosthetic control increasingly incorporate adaptive and task-dependent control strategies, highlighting the importance of accurately characterizing locomotor coordination across conditions [34,35,36]. However, the present results indicate that coordination geometry differs substantially between walking, stair negotiation, and constrained gait conditions. Beyond assistive technologies, these findings may also have clinical relevance, as altered inter-joint coordination has been associated with impaired balance control, compensatory gait strategies, and increased fall risk in neurological and musculoskeletal populations [37,38,39]. In this context, coordination geometry may provide a compact means of quantifying how inter-joint control strategies are altered in pathological gait or destabilized under challenging locomotor conditions. Recent advances in human-in-the-loop optimization and adaptive control of wearable robots highlight the importance of capturing user- and task-specific dynamics [34,35,36]. Incorporating coordination-level descriptors, such as loop magnitude, orientation, and anisotropy, may provide complementary coordination-level features for future studies investigating task-aware locomotor control and movement classification that adapt to changing locomotor demands. Furthermore, the low-dimensional nature of moment–moment representations makes them well suited for integration with data-driven approaches for movement classification and prediction. Recent work has demonstrated the potential of machine learning methods to capture and predict human movement dynamics in compact state-space representations [40], suggesting that coordination-based features may provide a powerful basis for real-time detection of locomotor states and transitions.

### 4.6. Limitations

Several limitations should be considered when interpreting these findings. First, joint moments were normalized within stride to isolate coordination structure, which removes information about absolute torque magnitude. While this approach is appropriate for studying coordination topology, future work could examine how coordination geometry interacts with absolute mechanical output. Second, the analysis was restricted to healthy individuals. It remains to be determined whether similar patterns of coordination reorganization are observed in clinical populations, where altered neuromechanical control may lead to different coordination geometries. Finally, PCA-based orientation measures may be less stable in conditions with low anisotropy. However, the dominant coordination patterns observed in the present study were robust across subjects and tasks, suggesting that the reported effects reflect consistent features of locomotor coordination.

## 5. Conclusions

Locomotor tasks induce structured, task-specific reorganization of ankle–knee mechanical coordination. Rather than reflecting a strictly invariant coordination template, inter-joint torque coupling adapts through systematic changes in coordination geometry. By representing coordination as a trajectory in moment–moment phase space, this approach provides a compact and interpretable framework for understanding how mechanical and functional demands shape locomotor behavior.

## Figures and Tables

**Table 1:** Summary of dataset composition and walking speed characteristics across locomotor tasks. Number of subjects and analyzed strides for each task condition. Speed statistics (median, first quartile Q1, third quartile Q3, and interquartile range IQR) are reported for stride-level walking speed within each task. Walking trials were filtered to retain natural-speed walking (±10% of each participant’s median walking speed), whereas other task conditions were analyzed without speed filtering.

Task	N Subjects	N Strides	Median Speed (m/s)	Q1 (m/s)	Q3 (m/s)	IQR (m/s)
Walking	49	331	1.261	1.133	1.306	0.173
ToeWalking	47	278	1.054	0.901	1.159	0.257
HeelWalking	45	178	0.713	0.625	0.836	0.211
StepUp	43	169	0.49	0.464	0.535	0.072
StepDown	43	146	0.494	0.455	0.561	0.105

**Table 2:** Linear mixed-effects model results and planned contrasts for ankle–knee coordination metrics across locomotor tasks. Linear mixed-effects model results testing the effect of locomotor task on ankle–knee coordination metrics. Models included task as a fixed factor and subject as a random intercept. Significant task effects were observed for loop magnitude, principal axis orientation, anisotropy, and axis rotation relative to walking.

Outcome	F	Num DF	Den DF	*p*
Loop magnitude|Area|	73.78278	4	222	1.11 × 10^−39^
PC1 angle	36.40173	4	222	2.19 × 10^−23^
Anisotropy	54.87074	4	222	4.07 × 10^−32^
ΔPC1 orientation (relative to walking)	2.829259	4	218	0.025643

**Table 3:** Planned pairwise contrasts comparing each locomotor task with natural walking. Planned pairwise contrasts comparing each locomotor task against natural-speed walking for ankle–knee coordination metrics. Estimates represent the difference relative to walking. *p*-values were adjusted using the Holm correction for multiple comparisons. Positive estimates indicate larger values than walking.

Loop Magnitude (|Area|)						
Contrast	Estimate	SE	t	DF	*p* (Holm)	95% CI
Walking vs. ToeWalking	3.446	0.22	15.66	222	3.91 × 10^−37^	[3.01, 3.88]
Walking vs. HeelWalking	1.355	0.223	6.08	222	1.02 × 10^−8^	[0.92, 1.79]
Walking vs. StepUp	0.814	0.226	3.61	222	3.82 × 10^−4^	[0.37, 1.26]
Walking vs. StepDown	2.406	0.226	10.67	222	3.09 × 10^−21^	[1.96, 2.85]
Anisotropy						
Contrast	Estimate	SE	t	DF	*p* (Holm)	95% CI
Walking vs. ToeWalking	0.245	0.038	6.46	222	1.31 × 10^−9^	[0.17, 0.32]
Walking vs. HeelWalking	0.402	0.038	10.49	222	1.08 × 10^−20^	[0.33, 0.48]
Walking vs. StepUp	0.023	0.039	0.59	222	0.556	[−0.05, 0.10]
Walking vs. StepDown	0.436	0.039	11.24	222	6.95 × 10^−23^	[0.36, 0.51]
ΔPC1 orientation (relative to walking)						
Contrast	Estimate (deg)	SE	t	DF	*p* (Holm)	95% CI
Walking vs. ToeWalking	−15.01	9.11	−1.65	218	0.303	[−32.96, 2.95]
Walking vs. HeelWalking	−7.31	9.23	−0.79	218	0.768	[−25.50, 10.88]
Walking vs. StepUp	−8.17	9.36	−0.87	218	0.768	[−26.61, 10.28]
Walking vs. StepDown	−29.84	9.36	−3.19	218	0.0066	[−48.28, −11.39]

Loop closure and circularity checks are reported in Appendix A.

**Figure A1:**
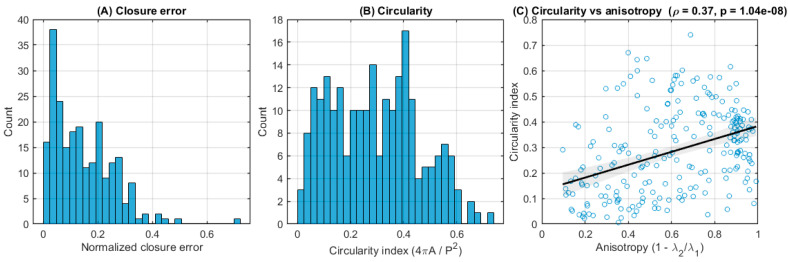
Loop validity checks and geometric relationship between circularity and anisotropy. (**A**) Distribution of normalized closure error for ankle–knee moment–moment loops, indicating that most trajectories formed near-closed loops over the analyzed stride. (**B**) Distribution of the circularity index (4πA/P^2^), describing the geometric compactness of the loops. (**C**) Relationship between circularity and PCA-based anisotropy, showing a moderate positive association (Spearman ρ = 0.37, *p* = 1.04 × 10^−8^). The line indicates a robust linear fit with 95% confidence band.

## Data Availability

The original data presented in the study are openly available in https://doi.org/10.6084/m9.figshare.11112980. The original contributions presented in this study are included in the Appendix A. Further inquiries can be directed to the corresponding author.

## Abbreviations

The following abbreviations are used in this manuscript:

Abbreviation	Definition
PCA	Principal Component Analysis
PC1	First Principal Component
PC2	Second Principal Component
GRF	Ground Reaction Force
CI	Confidence Interval
SE	Standard Error
IQR	Interquartile Range
FC_n_	Normalized Foot Contact

## Appendix A. Validation of Moment–Moment Loop Geometry

Ankle–knee coordination was analyzed using moment–moment phase space trajectories constructed from stride-normalized joint moments. For each stride, ankle and knee flexion moments were first extracted and time-normalized to 101 samples representing 0–100% of the gait cycle. To allow comparisons across participants and tasks, joint moments were z-scored within each stride prior to computing geometric loop metrics.

### Appendix A.1. Loop Area

The magnitude of the ankle–knee coordination loop was quantified using the area enclosed by the moment–moment trajectory. Loop area was computed using the polygon shoelace formula applied to the closed trajectory:

(A1) $$A=\frac{1}{2}\sum_{i=1}^{N}\left(x_{i}y_{i+1}-x_{i+1}y_{i}\right)$$

where x and y represent the normalized ankle and knee moments, respectively. The first sample of the trajectory was appended to the end of the sequence to ensure loop closure during computation. Both signed and absolute loop areas were retained. The signed area reflects the direction of loop traversal, whereas the absolute area represents the magnitude of joint interaction.

### Appendix A.2. Loop Perimeter

Loop perimeter was estimated as the cumulative Euclidean distance between successive samples along the trajectory:

(A2) $$P=\sum_{i=1}^{N-1}\sqrt{(x_{i+1}-x_{i}{)}^{2}+(y_{i+1}-y_{i}{)}^{2}}$$

This polygonal path-length approximation provides a robust estimate of trajectory perimeter for discretely sampled loops.

### Appendix A.3. Principal Component Orientation and Anisotropy

The dominant orientation of the moment–moment loop was quantified using principal component analysis (PCA). PCA was applied to the two-dimensional trajectory defined by ankle and knee moments, yielding eigenvectors and eigenvalues describing the principal axes of variance.

The orientation of the first principal component (PC1) relative to the ankle moment axis was computed as:

(A3) $$\theta =arctan(\frac{v_{y}}{v_{x}})$$

where $v_{x}$ and $v_{y}$ are the components of the first eigenvector. Because the coordination axis is directionally symmetric, the orientation was treated as an axial variable and mapped to the range 0–180°.

The degree of directional dominance in the loop was quantified using an anisotropy index derived from the PCA eigenvalues:

(A4) $$\mathrm{Anisotropy}=1-\frac{\lambda_{2}}{\lambda_{1}}$$

where $\lambda_{1}$ and $\lambda_{2}$ represent the first and second eigenvalues, respectively. This metric ranges from 0 for isotropic loops to values approaching 1 for strongly elongated loops dominated by a single coordination axis.

### Appendix A.4. Circularity Index

Loop compactness was quantified using a circularity index:

(A5) $$CI=\frac{4\pi A}{P^{2}}$$

where A is loop area and P is loop perimeter. Circularity approaches 1 for perfectly circular loops and decreases toward 0 as loop geometry becomes increasingly elongated.

### Appendix A.5. Loop Closure Error

To assess the validity of the extracted loops, a closure error metric was computed as the Euclidean distance between the first and last samples of the trajectory:

(A6) $$E_{closure}=\sqrt{(x_{end}-x_{start}{)}^{2}+(y_{end}-y_{start}{)}^{2}}$$

Closure error was normalized relative to loop scale using the square root of loop area. This metric quantifies the degree to which discretised trajectories form closed loops over the analysed stride.

### Appendix A.6. Loop Validity Metrics

To verify the validity and geometric consistency of the extracted ankle–knee moment–moment loops, several quality and shape metrics were evaluated across all analyzed strides (Figure A1).

First, the normalized loop closure error was quantified to assess the degree to which the first and last samples of each trajectory coincided. Closure errors were generally small relative to loop scale, with the majority of values below 0.2 and only a small number of larger values extending toward approximately 0.7 (Figure A1A). These results confirm that the extracted trajectories formed near-closed loops, indicating consistent stride segmentation and stable moment–moment trajectories across the dataset.

Second, the circularity index of the loops was computed to characterize overall geometric compactness. Circularity values spanned a broad range (approximately 0.05–0.75), with most loops clustering between 0.2 and 0.5 (Figure A1B). This distribution indicates that ankle–knee coordination loops were typically elliptical rather than circular, consistent with directional coupling between the two joint moments.

Finally, the relationship between circularity and the PCA-based anisotropy metric used in the main analysis was examined (Figure A1C). Circularity exhibited a moderate positive association with anisotropy (Spearman ρ = 0.37, *p* = 1.04 × 10^−8^). This relationship indicates that loops exhibiting stronger directional dominance in the principal component structure tended to display more regular geometric structure in the moment–moment plane.

Together, these analyses confirm that the extracted loops were geometrically well defined and that the circularity metric behaves consistently with the PCA-based anisotropy measure used to characterize loop shape.
